# Modified homotopy perturbation method for solving hypersingular integral equations of the first kind

**DOI:** 10.1186/s40064-016-3070-z

**Published:** 2016-09-01

**Authors:** Z. K. Eshkuvatov, F. S. Zulkarnain, N. M. A. Nik Long, Z. Muminov

**Affiliations:** 1 Faculty of Science and Technology, Universiti Sains Islam Malaysia (USIM), Negeri Sembilan, Malaysia; 2 Department of Mathematics, Faculty of Science, Universiti Putra Malaysia (UPM), Selangor, Malaysia; 3 Institute for Mathematical Research, Universiti Putra Malaysia (UPM), Selangor, Malaysia; 4 Faculty of Mathematics and Mechanics, Samarkand State University, Samarkand, Uzbekistan

**Keywords:** Homotopy perturbation method, Hypersingular integral equation, Integral equation, 45E05 (Integral equations with kernels of Cauchy type ), 30E20 (Integration, integrals of Cauchy type, integral representations of analytic functions)

## Abstract

Modified homotopy perturbation method (HPM) was used to solve the hypersingular integral equations (HSIEs) of the first kind on the interval [−1,1] with the assumption that the kernel of the hypersingular integral is constant on the diagonal of the domain. Existence of inverse of hypersingular integral operator leads to the convergence of HPM in certain cases. Modified HPM and its norm convergence are obtained in Hilbert space. Comparisons between modified HPM, standard HPM, Bernstein polynomials approach Mandal and Bhattacharya (Appl Math Comput 190:1707−1716, 2007), Chebyshev expansion method Mahiub et al. (Int J Pure Appl Math 69(3):265–274, 2011) and reproducing kernel Chen and Zhou (Appl Math Lett 24:636–641, 2011) are made by solving five examples. Theoretical and practical examples revealed that the modified HPM dominates the standard HPM and others. Finally, it is found that the modified HPM is exact, if the solution of the problem is a product of weights and polynomial functions. For rational solution the absolute error decreases very fast by increasing the number of collocation points.

## Background

Hypersingular integral equations (HSIEs) arise a variety of mixed boundary value problems in mathematical physics such as water wave scattering (Kanoria and Mandal 2002), radiation problems involving thin submerged plates (Parsons and Martin 1994) and fracture mechanics (Chan et al. 2003; Nik Long and Eshkuvatov 2009). Chen and Zhou (2011) have solved HSIE using the improvement of reproducing kernel method. Golberg (1987) obtained the approximate solution of HSIEs using Galerkin and collacation method and discuss their convergence. Spline collocations method has also been used to solve linear HSIE of the first kind and nonlinear HSIE of the second kind in Boykov et al. (2010, 2014) respectively. Projection method with Chebyshev polynomials were discussed to solve the singular and hypersingular integral equation of the first kind in Eshkuvatov et al. (2009), Mahiub et al. (2011) respectively.

Homotopy perturbation method (HPM) has been used for a wide range of problems He (1999, 2000), Khan and Wu (2011), Madani et al. (2011), Ramos (2008), Słota (2010), Jafari et al. (2010), Golbabai and Javidi (2007), Dehghan and Shakeri (2008), Ghasemi et al. (2007), Panda et al. (2015), Okayama et al. (2011), Panda (2013), Javidi and Golbabai (2009), Ghorbani and Saberi-Nadjafi (2006), Mohamad Nor et al. (2013). Particularly, He (1999, 2000) was pioneer of establishing HPM and used it to solve the linear and nonlinear differential equations. Khan and Wu (2011) used He’s polynomials to solve nonlinear problems. Madani et al. (2011) employed HPM together with Laplace transform for solving one-dimensional non-homogeneous partial differential equations with a variable coefficients. Other usage of HPM were finding the exact and approximate solutions of nonlinear ordinary differential equations (ODEs) (Ramos 2008), one-phase inverse Stefan problem (Słota 2010), linear and nonlinear integral equations (Jafari et al. 2010), the integro-differential equations (Golbabai and Javidi 2007; Dehghan and Shakeri 2008) and nonlinear Volterra–Fredholm integral equations Ghasemi et al. (2007). In Panda et al. (2015), a modified Lagrange approach is presented to obtain approximate numerical solutions of Fredholm integral equations of the second kind. The error bound is explained by the aid of several illustrative examples. In Okayama et al. (2011), two improved versions of the Sinc-collocation scheme are presented. The first version is obtained by improving the scheme so that it becomes more practical, and natural from a theoretical view point. In the second version, the variable transformation employed in the original scheme, the tanh transformation, is replaced with the double exponential transformation. It is proved that the replacement improves the convergence rate drastically. Numerical examples which support the theoretical results are also given. In Panda (2013), some recently developed analytical methods namely; homotopy analysis method, homotopy perturbation method and modified homotopy perturbation method are applied successfully for solving strongly nonlinear oscillators. The analytical results obtained by using HAM are compared with those of HPM, mHPM.

To improve the efficiency of the HPM, a few modifications have been made by many researches. For instance, Javidi and Golbabai (2009) added the accelerating parameter to the perturbation equation for obtaining the approximate solution for nonlinear Fredholm integral equation. Ghorbani and Saberi-Nadjafi (2006) added a series of parameter and selective functions to HPM to find the semi-analytical solutions of nonlinear Fredholm and Volterra integral equations. Mohamad Nor et al. (2013) developed the new homotopy function using De Casteljau algorithms to solve the algebraic nonlinear problems.

Consider HSIE of the first kind

1 $$\begin{aligned} \frac{1}{\pi } = \!\!\!\!\!\!\!\int _{-1}^{1} \frac{K(x,t)}{(t-x)^2}\varphi (t)\,dt+\frac{1}{\pi } \int _{-1}^{1} L_1(x,t) \,\varphi (t)\,dt = f(x),\quad -1<x<1, \end{aligned}$$

where $\varphi (x)$ is the unknown function of *x* to be determined, *K*(*s*, *t*) and $L_1(s,t)$ are the square integrable kernels on $D=\{(s,t)\in \mathbb {R}^2|-1\le s, t\le 1\}$. Assume that *K*(*s*, *t*) is constant on the diagonal of the region, i.e.

2 $$\begin{aligned} K(x,t)=c_0+(t-x)K_1(x,t), \end{aligned}$$

where $c_0$ is a nonzero constant and $K_1(x,t)$ is square integrable kernel of the form

$$\begin{aligned} K_1(x,t)=Q(x)+(t-x)Q_1(x,t), \end{aligned}$$

*Q*(*x*) is smooth function and $Q_1(x,t)$ is square integrable kernel.

The main objective is to find the bounded solution of Eq. (1). We search a solution in the form

3 $$\begin{aligned} \varphi (x)=\sqrt{1-x^2}u(x). \end{aligned}$$

Substituting Eqs. (2) and (3) into (1) yields

4 $$\begin{aligned}&\displaystyle \frac{c_0}{\pi }= \!\!\!\!\!\!\! \int _{-1}^{1} \frac{\sqrt{1-t^2}}{(t-x)^2}\,u(t)\,dt + \frac{Q(x)}{\pi }-\!\!\!\!\!\!\int _{-1}^{1} \frac{\sqrt{1-t^2}}{t-x}\,u(t)\,dt \nonumber \\&\quad \displaystyle + \frac{1}{\pi } \int _{-1}^{1} \sqrt{1-t^2} L(x,t) \,u(t) \,dt = f(x),\quad -1<x<1, \end{aligned}$$

where $$L(x,t)=Q_1(x,t)+L_1(x,t)$$.

Let us rewrite Eq. (4) in operator form

5 $$\begin{aligned} Hu+Cu+Lu =f, \end{aligned}$$

where

$$\begin{aligned} \displaystyle Hu(x)&=\frac{c_0}{\pi }=\!\!\!\!\!\!\!\int _{-1}^{1} \frac{\sqrt{1-t^2}}{(x-t)^2}\,u(t)\, dt, \\ \displaystyle Cu(x)&=\frac{Q(x)}{\pi }-\!\!\!\!\!\!\!\int _{-1}^{1} \frac{\sqrt{1-t^2}}{t-x}\,u(t)\, dt, \\ \displaystyle Lu(x)&=\frac{1}{\pi } \int _{-1}^{1} \sqrt{1-t^2} \,L(x,t) \,u\,dt. \end{aligned}$$

In this paper, the standard (convex) HPM and the modification of improved HPM (in short modified HPM) are utilized to find the bounded approximate solution of HSIEs (4). Norm convergence for both HPM and modified HPM are proved.

The structure of this paper is arranged as follows. In “Hilbert spaces and operators” section, related information regarding to the Hilbert spaces and operators theory are given. Description of standard HPM and modified HPM are presented in “HPM and modified HPM for HSIEs” section. Norm convergence of both standard HPM and modified HPM are proved in “Convergence of the methods” section. Implementation of modified HPM and its comparisons with others are shown in “Numerical examples” section. Finally, “Conclusion” section is for the conclusion.

## Hilbert spaces and operators

Let us consider some well known facts concerning the operator *H* in Eq. (5). Let

6 $$\begin{aligned} U_n(\theta )=\frac{\sin \left[ (n+1)\,\theta \right] }{\sin \theta }, \qquad \theta =\cos ^{-1}x, \qquad n=0,1,2,\ldots , \end{aligned}$$

denote the Chebyshev polynomials of the second kind, and

7 $$\begin{aligned} \phi _n=\sqrt{\frac{2}{\pi }}\,U_n, \end{aligned}$$

is normalized Chebyshev polynomials of the second kind

$$\begin{aligned} \int _{-1}^{1}\sqrt{1-t^2}\,\phi _n^2(t) \,dt=1. \end{aligned}$$

It is well known that the hypersingular operator $H_g$ can be considered as the differential Cauchy operator i.e.,

8 $$\begin{aligned} H_gu=\frac{1}{\pi }=\!\!\!\!\!\!\int _{-1}^{1}\frac{\sqrt{1-t^{2}}u(t)}{(t-x)^2}\,dt= \frac{d}{dx}\frac{1}{\pi }-\!\!\!\!\!\!\int _{-1}^{1}\frac{\sqrt{1-t^{2}}u(t)}{t-x} dt = \frac{d}{dx}C_gu. \end{aligned}$$

as well as acting operator $C_g$ for Chebyshev polynomials of second kind yields

9 $$\begin{aligned} C_gU_n(x)=\frac{1}{\pi }-\!\!\!\!\!\!\int _{-1}^{1}\frac{\sqrt{1-t^{2}}U_n(t)}{t-x} dt= -T_{n+1}(x), \end{aligned}$$

where $T_{n+1}(x)$ is the Chebyshev polynomial of the first kind.

It can easily be shown from (7), (8), (9) and $T_{n+1}'(x)=(n+1)U_n(x)$ that

10 $$\begin{aligned} H\phi _n(x)=-c_0(n+1)\,\phi _n(x), \end{aligned}$$

11 $$\begin{aligned} C\phi _n(x)&=-\frac{Q(x)}{2}\Bigl (\phi _{n+1}(x) - \phi _{n-1}(x)\Bigr ), \quad n=0,1,\ldots , \end{aligned}$$

where $\phi _{-1}(x)=0$. Note that Eqs. (10) and (11) are crucial to the rest of our analysis.

Let $L(\rho )$ denotes the space of square integrable real valued function with respect to $\rho (x)=\sqrt{1-x^2}$. The inner product on $L(\rho )$ is given by

$$\begin{aligned} \langle u,v \rangle _\rho =\int _{-1}^{1} \rho (t)\,u(t)\,v(t)\,dt, \end{aligned}$$

and $\Vert u\Vert _\rho =\sqrt{\langle u,v \rangle _\rho }$ denotes the norm.

The set $\{\phi _k\}_{k=0}^{\infty }$ is a complete orthonormal basis for $L(\rho )$, so that if $u\in L(\rho )$ then

$$\begin{aligned} u=\sum _{k=0}^{\infty }\langle u,\phi _k \rangle _\rho \phi _k, \end{aligned}$$

where the sum converges in $L(\rho )$. In addition, the norm of *u* satisfies the Parseval’s equality

$$\begin{aligned} \Vert u\Vert _\rho ^2=\sum _{k=0}^{\infty }\langle u,\phi _k \rangle _\rho ^2. \end{aligned}$$

We will need the subspace of $L(\rho )$ which is consisting of all *u* such that

12 $$\begin{aligned} \sum _{k=0}^{\infty }(k+1)^2 \langle u,\phi _k \rangle _\rho ^2<\infty . \end{aligned}$$

All functions satisfying (12) is denoted by $L_1(\rho )$ and it can be made into Hilbert space if the inner product of $u \in L_1(\rho )$ and $v \in L_1(\rho )$ are defined by

$$\begin{aligned} \langle u,v \rangle _1=\sum _{k=0}^{\infty } (k+1)^2 \langle u,\phi _k \rangle _\rho \langle v,\phi _k \rangle _\rho . \end{aligned}$$

The norm of $u \in L_1(\rho )$ is given by

13 $$\begin{aligned} \Vert u\Vert _1^2=\sum _{k=0}^{\infty }(k+1)^2 \langle u,\phi _k \rangle _\rho ^2. \end{aligned}$$

We extend the operator *H* defined by (5) as a bounded operator from $L_1(\rho )$ to $L(\rho )$ by defining

$$\begin{aligned} H u=c_0 \sum _{k=0}^{\infty }\langle u,\phi _k \rangle _\rho H\phi _k=c_0\sum _{k=0}^{\infty }\langle u,\phi _k \rangle _\rho (-(k+1))\phi _k, \end{aligned}$$

and observe that

$$\begin{aligned} \Vert H u\Vert _\rho ^2=c_0^2\sum _{k=0}^{\infty }(k+1)^2\langle u,\phi _k \rangle _\rho ^2 =c_0^2 \Vert u\Vert _1^2. \end{aligned}$$

It is not hard to show that $H^{-1}:L_1(\rho )\rightarrow L(\rho )$ exist and is given by

14 $$\begin{aligned} H^{-1}u=\frac{1}{c_0}\sum _{k=0}^{\infty }\left( -\frac{\langle u,\phi _k > _\rho }{k+1}\right) \phi _k, \end{aligned}$$

hence *H* is invertible Golberg (1987).

### **Lemma 1**

*The norm of operator*$H^{-1}: L_1(\rho )\rightarrow L(\rho )$*is*

15 $$\begin{aligned} \Vert H^{-1}\Vert =\frac{1}{|c_0|}. \end{aligned}$$

### *Proof*

Assume that $H^{-1}u=v$. On the other hand

16 $$\begin{aligned} \langle v,\phi _k \rangle \,=\,\langle H^{-1}u,\phi _k \rangle \,=\,-\frac{1}{c_0}\dfrac{\langle u,\phi _k \rangle}{k+1}. \end{aligned}$$

Since $v\in L_1(\rho )$ and due to (16) we have

$$\begin{aligned} \Vert v\Vert ^2_1&=\sum _{k=0}^{\infty } (k+1)^2 \,(\langle v,\phi _k \rangle)^2 \\&=\frac{1}{c_0^2}\sum _{k=0}^{\infty }\, ( \langle u,\phi _k \rangle )^2 \\&=\frac{1}{c_0^2} \,\Vert u\Vert ^2_{\rho }. \end{aligned}$$

Therefore

$$\begin{aligned} \Vert H^{-1}u\Vert _1&=\frac{1}{|c_0|}\Vert u\Vert _\rho . \end{aligned}$$

By the norm definition of operator, we obtain

17 $$\begin{aligned} \left\| H^{-1}\right\| =\sup _{\begin{array}{c} u\in L_1(\rho )\\ \Vert u\Vert _{\rho }\le 1 \end{array} } \left\| H^{-1}u\right\| _1 = \dfrac{1}{|c_0|}\,\sup _{\Vert u\Vert _{\rho }\le 1}\Vert u\Vert _{\rho }= \frac{1}{|c_0|}. \end{aligned}$$

$$\square$$

### Some facts from operators theory

#### **Lemma 2**

*LetA*, *B be operators acting in Hilbert space. IfAis bounded andBis compact then the productsABandBAare compact*.

Lemma 2 is proven in Reed and Simon (1980, Theorem VI.12, pp. 200).

#### **Lemma 3**

*The operators*$C: L_{1}(\rho ) \rightarrow L(\rho )$*and*$H^{-1}C: L_{1}(\rho ) \rightarrow L_{1}(\rho )$*are compact*.

#### *Proof*

Let us define operators $T_r, \ \ T_l: \, L_{1}(\rho ) \rightarrow L(\rho )$ as

18 $$\begin{aligned} T_ru = \sum _{k=0}^\infty \langle u, \phi _k\rangle _\rho \phi _{k+1}, \nonumber \\ \quad T_lu = \sum _{k=1}^\infty \langle u, \phi _k\rangle _\rho \phi _{k-1}. \end{aligned}$$

These operators are bounded from $L(\rho ) \rightarrow L(\rho )$. Moreover, boundedness and the compactly embeddability of $T_r$ and $T_l$ from $L_{1}(\rho )$ to $L(\rho )$ (Berthold Berthold et al. (1992, Conclusion 2.3)) implies the compactness of $T_r$ and $T_l$. From (11) and (18) it follows that

19 $$\begin{aligned} Cu(x)=\frac{Q(x)}{2}(T_r-T_l)u(x). \end{aligned}$$

Since operators $T_r$ and $T_l$ are compact, its linear combinations is also compact i.e. $T_r-T_l: \, L_{1}(\rho ) \rightarrow L(\rho )$. As we know *Q*(*x*) is a continues function on the closed interval $[-1,1]$ and $T_r-T_l$ is compact, their product *C* is also compact by Lemma 2. On the other hand $H^{-1}$ is unitary and *C* is compact due to Lemma 2. Hence, operator $H^{-1}C$ is compact.$\square$

Since operators *C* and *L* are compact then $C+L:L_{1}(\rho ) \rightarrow L_{1}(\rho )$ is also compact. We know that $H^{-1}(C+L):L_1(\rho )\rightarrow L_1(\rho )$ is a compact operator. Due to the Fredholm theorem Reed and Simon (1980, Theorem VI.14) the inverse operator $(I+\lambda H^{-1}(C+L) )^{-1}$ of the operator function $I+\lambda H^{-1}(C+L), \lambda \in \mathbb C$, exists for all $\lambda$ in $\mathbb C\setminus C_1$, where $C_1$ is a discrete subset of $\mathbb C$ (i.e. a set $C_1$ has no limit points in $\mathbb C$) and for $\lambda \in C_1$ the null space $N(I+\lambda H^{-1}(C+L))$ is finite, that is $z=-\lambda ^{-1}$ is the eigenvalue of $H^{-1}(C+L)$ with finite multiplicity. These facts allows us to suppose the following

#### **Assumption 4**

$\lambda =1$ does not belong to $C_1$, i.e. $N(I+\lambda H^{-1}(C+L))=\{0\}$.

#### **Lemma 5**

*Let the Assumption 4 is satisfied, then the operator*$H+C+L$*is invertible, and the main Eq.* (5) has a unique solution.

#### *Proof*

Since *H* is invertible we get the relation

$$\begin{aligned} H+C+L=H(I+\lambda H^{-1}(C+L)), \quad (I+\lambda H^{-1}(C+L)):L_1(\rho )\rightarrow L_1(\rho ) , \end{aligned}$$

which gives us the fact that $H+C+L$ is invertible iff $I+\lambda H^{-1}(C+L)$ is invertible. Then due to Assumption 4 the operator $H+C+L$ is invertible.$\square$

#### **Assumption 6**

Assume that $S=H+C+L$ where *H*, *C*, *L* are defined by (5) is an invertible operator such that

20 $$\begin{aligned} N(H+C+L)=0, \end{aligned}$$

where *N*(*A*) is a nullspace of *A*.

Let $P_n:L(\rho )\rightarrow L(\rho )$ be the orthogonal projection onto the subspace spaned by $\{ \phi _0,\phi _1,\,\ldots,\,\phi _n\}$ and

$$\begin{aligned} L_n = P_n L. \end{aligned}$$

#### **Lemma 7**

*If* (20) *holds then*

$$\begin{aligned} \Vert \tilde{L}\Vert =\Vert L-L_n\Vert <\frac{\varepsilon }{\Vert \tilde{S}\Vert }, \end{aligned}$$

*and*$\tilde{S}=(H+C+L_n)$*exist and invertible operator*.

#### *Proof*

Since *L* is compact operator then $\tilde{L}\underset{n\rightarrow \infty }{\longrightarrow } 0$ i.e. for $\forall \varepsilon >0$, there exists $n_0$ such that $n\ge n_0$ implies

$$\begin{aligned} \Vert \tilde{L}\Vert =\Vert L-L_n\Vert <\frac{\varepsilon }{\Vert \tilde{S}\Vert }. \end{aligned}$$

Due to invertibility of $S=H+C+L$ and $\tilde{L}=L-L_n \underset{n\rightarrow \infty }{\longrightarrow } 0$ we obtain

$$\begin{aligned} \Vert (H+C+L)^{-1}\tilde{L}\Vert \le 1. \end{aligned}$$

Hence $I-(H+C+L)^{-1}\tilde{L}$ is invertible and

$$\begin{aligned} (I-(H+C+L)^{-1}\tilde{L})^{-1}(H+C+L)^{-1} = (H+C+L_N)^{-1} \end{aligned}$$

due to Lemma 2 operator $\tilde{S}=H+C+L_n$ is invertible.$\square$

## HPM and modified HPM for HSIEs

### HPM for HSIE

We present the application of standard HPM for solving hypersingular integral equations of the first kind (5). The perturbation scheme in convex homotopy form is given by

21 $$\begin{aligned} H^*(v,p)=(1-p)(Hv-u_0)+p\,(Hv+Cv+Lv-f), \end{aligned}$$

where $p\in [0,1]$ is homotopy parameter. For $p = 0$ the solution of the operator equation $H^*(v, 0) = 0$ is equivalent to the solution of a trivial problem $Hv(x)-u_{0}(x)=0$. For $p=1$ the equation $H^*(v,1)=0$ leads to the solution of Eq. (5).

The solution of operator equation $H^*(v,p)=0$ is searched in the form of power series

22 $$\begin{aligned} v(x)=\sum _{k=0}^{\infty }p^{k}v_{k}(x). \end{aligned}$$

We assume that the series (22) possesses a radius of convergence not smaller than 1. Substituting (22) into (5) yelds

23 $$\begin{aligned} H\left( \sum _{k=0}^{\infty }p^{k}v_{k}(x)\right) =u_0+p\left[ f-C\left( \sum _{k=0}^{\infty }p^{k}v_{k}(x)\right) - L\left( \sum _{k=0}^{\infty }p^{k}v_{k}(x)\right) -u_0\right] . \end{aligned}$$

Existence of $H^{-1}$ and equating the coefficients of like powers of *p* in Eq. (23), leads to the following iterations

24 $$\begin{aligned} v_0&=H^{-1}(u_0),\nonumber \\ v_1&=H^{-1}(f-u_0-C v_0-L v_0),\nonumber \\ v_k&=H^{-1}(-C v_{k-1}-L v_{k-1}), \ \ k\ge 2. \end{aligned}$$

By computing the iterations $v_k$ in Eq. (24), we can find semi-analytical solution as follows

25 $$\begin{aligned} \varphi (x)=\sqrt{1-x^2}\Bigl (v_0(x)+v_1(x)+\cdots \Bigr ) \end{aligned}$$

Approximate solution can be computed by

26 $$\begin{aligned}&\varphi _N(x)=\sqrt{1-x^2}\Bigl (v_0(x)+v_1(x)+\cdots +v_N(x) \Bigr ) \nonumber \\&\qquad \quad = \sqrt{1-x^2} \tilde{v}_N, \end{aligned}$$

where

27 $$\begin{aligned} \tilde{v}_N=v_0(x)+v_1(x)+\cdots +v_N(x). \end{aligned}$$

### Modified HPM for HSIEs

Let us rewrite Eq. (5) in the equivalent form

28 $$\begin{aligned} \tilde{S}u+\tilde{L}u=f, \end{aligned}$$

where

$$\begin{aligned}&S=H+C+L, \ \ \tilde{S}=H+C+L_n \; \text {with} \ \ \Vert S-\tilde{S}\Vert \underset{n\rightarrow \infty }{\longrightarrow } 0, \\&\tilde{L}=L-L_n, \; \text {with}\; \ \ \Vert \tilde{L}\Vert =\Vert L-L_n\Vert \underset{n\rightarrow \infty }{\longrightarrow } 0. \end{aligned}$$

Modified HPM for Eq. (28) is constructed as

$$\begin{aligned} H^*(v,p)&=(1-p)\left( \tilde{S}v-\sum _{j=0}^{m}\alpha _j\,g_j(x) \right) +p\,(\tilde{S}v+\tilde{L}v-f),\\&=\tilde{S}v-\sum _{j=0}^{m}\alpha _j\,g_j(x)+p \left( \tilde{L}v + \sum _{j=0}^{m}\alpha _j\,g_j(x)-f\right) . \end{aligned}$$

Equating $H^*(v,p)=0$ leads to

29 $$\begin{aligned} \tilde{S}v=\sum _{j=0}^{m}\alpha _j\,g_j(x)-p \left( \tilde{L}v + \sum _{j=0}^{m}\alpha _j\,g_j(x)-f\right) . \end{aligned}$$

Substituting series solution (22) into (29) yields

$$\begin{aligned} \tilde{S}\left( \sum _{k=0}^{\infty }p^{k}v_{k}(x)\right) =\sum _{j=0}^{m}\alpha _j\,g_j(x)-p \left[ \tilde{L}\left( \sum _{k=0}^{\infty }p^{k}v_{k}(x)\right) +\sum _{j=0}^{m}\alpha _j\,g_j(x)-f \right] . \end{aligned}$$

Equating both sides to the like power of *p* gives

30 $$\begin{aligned} \tilde{S}(v_0)&=\sum _{j=0}^{m}\alpha _j\,g_j(x),\nonumber \\ \tilde{S}(v_1)&=-\tilde{L}(v_0)+f-\sum _{j=0}^{m}\alpha _j\,g_j(x),\nonumber \\ \tilde{S}(v_k)&=-\tilde{L}(v_{k-1}),\quad k=2,3,\ldots \end{aligned}$$

Since $\tilde{S}$ is invertible by Lemma 7, we have

31 $$\begin{aligned} v_0&=\tilde{S}^{-1}\left( \sum _{j=0}^{m}\alpha _j\,g_j(x)\right) ,\nonumber \\ v_1&=\tilde{S}^{-1}\left( -\tilde{L}(v_0)+f-\sum _{j=0}^{m}\alpha _j\,g_j(x)\right) , \nonumber \\ v_k&=\tilde{S}^{-1}\left( -\tilde{L}(v_{k-1})\right) ,\quad k=2,3,\ldots \end{aligned}$$

Semi-analytical solution of Eq. (5) can be computed by (25).

#### *Remark*

Note that most cases of modified HPM, the unknown coefficients $\alpha _j$ of $v_0$ in the first equation of (31) are defined by equating the next iteration $v_1$ to be zero and it leads to $v_k=0, \ \ k \ge 2$ which implies two step method. In general, if $v_1\not = 0$ but $v_1^{(m)}\rightarrow 0$ as $m\rightarrow \infty$ then we can compute the next iteration $v_k, k\ge 2$. It effects to the next iteration $k\ge 2$ but the contribution to the solution of the problem will be very small therefore we can neglect it.

## Convergence of the methods

### Convergence of HPM

Let us consider HSIE (5) by adding parameter $\lambda$ of the form

32 $$\begin{aligned} Hu+\lambda (C+L)u=f. \end{aligned}$$

Standard HPM for Eq. (32) has the scheme

33 $$\begin{aligned} v_0&=H^{-1}(u_0),\nonumber \\ v_1&=H^{-1}(f-u_0-\lambda (C+L) v_0),\nonumber \\ v_k&=H^{-1}(-\lambda (C +L) v_{k-1}), \ \ k\ge 2. \end{aligned}$$

Since $H^{-1}$ exists, Eq. (33) is computable. The convergence of the method is given in the following theorem.

#### **Theorem 8**

*Let*$\displaystyle K(x,t)=c_0+(t-x)K_1(x,t)$*and*$K_1(x,t), \ \ L(s,t) \in C(D)$*and*$f \in C[-1,1]$*be continuous functions. In addition, if the following inequality*

34 $$\begin{aligned} |\lambda |\,\Vert C+L\Vert <|c_0| , \end{aligned}$$

*holds and initial guess*$$u_0(t)$$*is chosen as a continuous function for*$$t \in [-1, 1]$$, *then the series* (22) *is norm convergent to the exact solutionuon the interval*$$[-1,1] $$*for each*$$p =[0,1] $$.

#### *Proof*

Let $\Vert u_0\Vert _\rho =M$ and $\Vert f\Vert _\rho =M_1$. Based on (33) and Lemma 1 we have

$$\begin{aligned} \nonumber \Vert v_0\Vert _1&\le \Vert H^{-1}\Vert \,\Vert u_0\Vert _\rho =\dfrac{M}{|c_0|},\\ \Vert v_1\Vert _1&\le \Vert H^{-1}\Vert \,\Vert f-u_0-\lambda \,(C+L)\,v_0\Vert _\rho ,\\&\le \frac{c_0 M_1+c_0 M+|\lambda |\,\Vert C+L\Vert \,M}{|c_0|^2}= B,\\ \Vert v_2\Vert _1&\le \Vert H^{-1}\Vert \,\Vert \lambda \,(C +L)\,v_1\Vert _\rho ,\\&\le \dfrac{|\lambda |\,\Vert C+L\Vert \,B}{|c_0|}\\ \Vert v_k\Vert _1&\le \left( \dfrac{|\lambda |\,\Vert C+L\Vert }{|c_0|}\right) ^{k-1}\,B,\\&=\gamma _{1}^{k-1}\,B, \ \ k\ge 2. \end{aligned}$$

where

35 $$\begin{aligned} \gamma _{1}&=\dfrac{|\lambda |\,\Vert C+L\Vert }{|c_0|} \end{aligned}$$

36 $$\begin{aligned} B&=\frac{1}{|c_0|^2}\Bigl [c_0 M_1+c_0 M+|\lambda |\,\Vert C+L\Vert \,M \Bigr ] . \end{aligned}$$

Assume that $\gamma _1<1$, then from (22) at $p=1$, we obtain

$$\begin{aligned} \Vert v\Vert _1&\le \left\| \sum _{k=0}^{\infty } v_k(x)\right\| _1\le \Vert v_0\Vert _1+\sum _{k=1}^{\infty } \Vert v_k\Vert _1,\\&\le M+\sum _{k=1}^{\infty } \gamma _{1}^{k-1}\,B=M+\frac{B}{1-\gamma _{1}}<\infty . \end{aligned}$$

Therefore, series (22) converges to the exact solution in the sense of norm $||\cdot ||_1$.$\square$

#### *Remark 9*

Note that in our case $\lambda = 1$ and the convergence of HPM can be established if and only if

$$\begin{aligned} \Vert C+L\Vert <|c_0|. \end{aligned}$$

It implies that HPM converges to the solution of HSIEs (5) in rare cases.

The first $N+1$ terms of series (22) as $p\rightarrow 1$ gives the approximate solution of the form

37 $$\begin{aligned} \tilde{v}_N(x)=\sum _{k=0}^{N}v_k(x). \end{aligned}$$

#### **Theorem 10**

*If*$\gamma _1<1$, *then the rate of convergence of the approximate solution*$\tilde{v}_N$*can be estimated by*

38 $$\begin{aligned} E_N\le \frac{\gamma _1^N}{1-\gamma _1}B, \end{aligned}$$

*where*$E_N=\Vert v(x)-\tilde{v}_N(x)\Vert _1$*andBis defined by* (36).

#### *Proof*

$$\begin{aligned} \Vert v(x)-\tilde{v}_N(x)\Vert _1&=\left\| \sum _{k=0}^{\infty }v_k(x)-\sum _{k=0}^{n}v_k(x)\right\| _1\\&=\left\| \sum _{k=N+1}^{\infty }v_k(x)\right\| _1 \le \sum _{k=N+1}^{\infty }\Vert v_k(x)\Vert _1\\&\quad \le \sum _{k=N+1}^{\infty }\gamma _1^{k-1} B\\&= \frac{\gamma _1^N}{1-\gamma _1}\,B. \end{aligned}$$

Since $\gamma _1<1$, the norm

$$\begin{aligned} ||v(x)-v_n(x)||_{1}\rightarrow 0 \end{aligned}$$

whenever $$n\rightarrow \infty $$.$$\square$$

### Convergence of modified HPM

Let us consider HSIE in the form of Eq. (28). If $v_1=0$ in (30), then $v_0$ satisfies $\tilde{S}v_0+\tilde{L}v_0=f$ and coincides with the exact solution. If $v_1 \ne 0$ then $v_1^{(m)}\rightarrow 0$ i.e. for any $\varepsilon$, there exists $m_0$, such that $m>m_0$ implies

39 $$\begin{aligned} \Vert v_1^{(m)}\Vert _1<\varepsilon . \end{aligned}$$

Let $\left\| \sum _{j=0}^{m}\alpha _j\,g_j(x))\right\| _\rho =M_2$ and $\Vert f\Vert _\rho =M_1$, then due to (31) and existence of $S^{-1}$ (Lemma 6) we obtain

$$\begin{aligned} \Vert v_0\Vert _1&=\left\| \tilde{S}^{-1}\left( \sum _{j=0}^{m}\alpha _j\,g_j(x) \right) \right\| _1 \\&\quad \le \Vert \tilde{S}^{-1}\Vert \,M_2,\\ \Vert v_1\Vert _1&=\left\| \tilde{S}^{-1}\left( -\tilde{L}(v_0)-\sum _{j=0}^{m}\alpha _j\,g_j(x)+f\right) \right\| _1 \\&\quad \le \varepsilon , \\ \Vert v_2\Vert _1&= \Bigl \Vert \tilde{S}^{-1}\left( \tilde{L}(v_1)\right) \Bigr \Vert _1\\&\quad \le \Bigl \Vert \tilde{S}^{-1}\Bigr \Vert \,\Bigl \Vert \tilde{L}\Bigr \Vert \,\Vert v_1\Vert _1\\&\quad \le \gamma _2 \,\varepsilon , \end{aligned}$$

where $\gamma _2=\Bigl \Vert \tilde{S}^{-1}\Bigr \Vert \,\Bigl \Vert \tilde{L}\Bigr \Vert$ and by continuing these procedure

40 $$\begin{aligned} \Vert v_k\Vert _1&=\Bigl \Vert \tilde{S}^{-1}\left( \tilde{L}(v_{k-1})\right) \Bigr \Vert _1 \nonumber \\&\quad \le \Bigl \Vert \tilde{S}^{-1}\Bigr \Vert ^{k-1}\,\Bigl \Vert \tilde{L}\Bigr \Vert ^{k-1}\,\Vert v_{1}\Vert _1 \nonumber \\&=\gamma _2^{k-1}\,\varepsilon . \end{aligned}$$

Due to $\Vert \tilde{L}\Vert =\Vert L-L_n\Vert \underset{n\rightarrow \infty }{\longrightarrow } 0$ it can be easily shown that $\gamma _2 <1$ for large enough *k* then

41 $$\begin{aligned} \Vert v\Vert _1& \le \left\| \sum _{k=0}^{\infty }v_{k}\right\| _1 = \Vert v_0\Vert _1+\left\| \sum _{k=1}^{\infty }v_{k}\right\| _1 \nonumber \\&\quad \le \left\| \tilde{S}^{-1}\right\| \,M_2+\sum _{k=1}^{\infty }\gamma _2^{k-1}\varepsilon \\&= \left\| \tilde{S}^{-1}\right\| \,M_1+\frac{\varepsilon }{1-\gamma _2}<\infty . \nonumber \end{aligned}$$

Thus, we have proved the following theorem

#### **Theorem 11**

*Let*$\displaystyle K(x,t)=c_0+(t-x)K_1(x,t)$*and*$K_1(x,t), \ \ L(s,t) \in C(D)$*and*$f \in C[-1,1]$*be continuous functions. In addition, if the following inequality*

42 $$\begin{aligned} \gamma _2 = \Vert \tilde{S}^{-1}\Vert \,\Vert \tilde{L}\Vert < 1 , \end{aligned}$$

*holds and selective functions*$$g_j(x), \ \ j=0,\ldots ,N$$*are chosen as a continuous function on the interval*$$[-1, 1]$$, *then the series solution* (22) is norm convergent to the exact solution $$\varphi (x)$$*on the interval*$$[-1,1] $$*for each*$$p =[0,1] $$.

#### *Remark 12*

Theorems 8 and 11 show the fact that the exact solution *u* belongs to $L_1(\rho )$. Then due to Berthold et al. (1992, Theorem 2.13) the function *u* belongs to $C^{(1)}(-1,1)$.

Approximate solution of Eq. (4) in series (37) can be estimated as follows.

#### **Theorem 13**

*Rate of convergence of approximate solution*$\tilde{v}_N$*can be estimated by*

43 $$\begin{aligned} E_N\le \frac{\upgamma _2^N}{1-\upgamma _2}\varepsilon , \end{aligned}$$

*where*$E_n=\Vert v(x)-\tilde{v}_N(x)\Vert$*and*$\varepsilon$*are defined by* (39) and $\gamma _2<1$.

#### *Proof*

$$\begin{aligned} \Vert v(x)-\tilde{v}_N(x)\Vert _1&=\left\| \sum _{k=0}^{\infty }v_k(x)-\sum _{k=0}^{N}v_k(x)\right\| _1\\&=\left\| \sum _{k=N+1}^{\infty }v_k(x)\right\| _1 \le \sum _{k=N+1}^{\infty }\Vert v_k(x)\Vert _1\\&\quad \le \sum _{k=N+1}^{\infty }\gamma _2^{k-1} \varepsilon \\&= \frac{\gamma _2^N}{1-\gamma _2}\,\varepsilon . \end{aligned}$$

$$\square$$

#### *Remark 14*

Since $\gamma _2 < 1$, the term $\displaystyle \frac{\gamma _2^N}{1-\gamma _2}\varepsilon \rightarrow 0$ as $N\rightarrow \infty$. Moreover, sufficiently small $\varepsilon$ gives the smaller error rate for $E_N$ in (43) than error $E_N$ in (38). This fact shows that the modified HPM is dominates the standard HPM.

## Numerical examples

### *Example 1*

(Mandal and Bhattacharya 2007). Consider HSIE (1) of the form

44 $$\begin{aligned} \frac{1}{\pi } =\!\!\!\!\!\!\int _{-1}^{1} \frac{\varphi (t)}{(x-t)^2}\, dt= 1,\quad -1<x<1. \end{aligned}$$

The exact solution of Eq. (44) is $\varphi (x)=-\sqrt{1-x^2}$ and $c_0=1, f(x)=1$.

*Solution* It is easy to find that Eq. (44) satisfied all conditions in Theorem 8. To apply HPM, we choose the initial guess as $u_0=\phi _0(x)$. Since $Cu=Lu\equiv 0$ and from (14) we can easily get

45 $$\begin{aligned} H^{-1}(\phi _j) = -\frac{1}{c_0}\frac{\phi _j}{j+1}. \end{aligned}$$

Referring to (24) and using (45), we obtain successive functions

$$\begin{aligned} v_0(x)&=H^{-1}(\phi _0)=-\phi _0, \\ v_1(x)&=H^{-1}(1-\phi _0)=\left( -1+ \phi _0\right) , \\ v_j(x)&=0. \ \ j=2,3,\ldots \end{aligned}$$

Since $v_2(x)=v_3(x)=\cdots =0$, the approximate solution of Eq. (44) is

46 $$\begin{aligned} \varphi (x)=\sqrt{1-x^2}(v_0(x)+v_1(x))=-\sqrt{1-x^2}, \end{aligned}$$

which is identical with exact solution.

For application of modified HPM to the Eq. (44), we do this following steps:

1. Let selective functions $g_j(x)=\phi _j(x), j=0, \ldots m$. Since $\tilde{S}=H, \ \ \tilde{L}\equiv 0$ for (44) we can use inverse operator (45). Based on the scheme (31) for $m=2$ we obtain,
  $$\begin{aligned} v_0(x)&=H^{-1}(\alpha _0\,\phi _0+\alpha _1\,\phi _1+\alpha _2\,\phi _2) = -\alpha _0\phi _0-\frac{\alpha _1}{2}\, \phi _1-\frac{\alpha _2}{3}\, \phi _2, \nonumber \\ v_1(x)&=H^{-1}(1- \alpha _0\,\phi _0 -\alpha _1\,\phi _1 -\alpha _2\,\phi _2) = -1 + \alpha _0\phi _0 +\frac{\alpha _1}{2}\,\phi _1 +\frac{\alpha _2}{3}\,\phi _2, \nonumber \\ v_k&= H^{-1}(0) \equiv 0, \, k=2,3, \ldots \end{aligned}$$
2. Since $v_k \equiv 0, \, k=2,3, \ldots$ we can easily find approximate solution as
  47 $$\begin{aligned} \varphi (x)=\sqrt{1-x^2}(v_0(x)+v_1(x))=-\sqrt{1-x^2}, \end{aligned}$$
  which coincides with exact solution.

Mandal and Bhattacharya (2007) consider the Eq. (44) and comparisons with HPM, modified HPM are summarized in Table 1.

**Table 1:** Errors of methods for Eq. (44)

*x*	Error in Mandal and Bhattacharya (2007)	Error of HPM	Error of modified HPM
−1	0	0	0
−0.5	1.3 × 10^−17^	0	0
0	0	0	0
0.5	7.8 × 10^−18^	0	0
1	0	0	0

### *Example 2*

Mahiub et al. (2011). Consider HSIE of the form

48 $$\begin{aligned} \frac{1}{\pi } =\!\!\!\!\!\!\int _{-1}^{1} \frac{\varphi (t)}{(x-t)^2}\, dt+\frac{1}{\pi } \int _{-1}^{1} \sin (x)t^4 \,\varphi (t)\, dt = -5(16 x^4-12x^2+1)-\frac{\sin (x)}{32},\quad -1<x<1, \end{aligned}$$

with exact solution $\varphi (x)=\displaystyle \sqrt{1-x^2}\,(16 x^4-12x^2+1)$.

*Solution* Conditions of the Theorem 8 does not hold for Example 2. Therefore we did comparisons between modified HPM and method given in Mahiub et al. (2011).

To solve the Eq. (48) by modified HPM we do the following steps:

1. Let us choose selective functions $g_j(x)=\phi _j(x), \, j=0,\ldots , m$ and kernel $L(x,t)=\sin (x)t^4$ in Eq. (48) be approximated by projection kernel $L_n(x,t)=\sum _{i=1}^{l}b_i(x)\phi _i(t)$. In this case $\tilde{L}u=Lu-L_nu \equiv 0$. Since $Cu\equiv 0, \ \ \tilde{L}u \equiv 0$ then $\tilde{S}=H+L$. From (30) it follows that
  49 $$\begin{aligned} (H+L)v_0&=\sum _{j=0}^{m}\alpha _j\,\phi _j(x),\nonumber \\ (H+L)v_1&=f-\sum _{j=0}^{m}\alpha _j\,\phi _j(x). \nonumber \\ (H+L)v_k&=-\tilde{L}(v_{k-1}) \equiv 0, \quad k=2,3, \ldots \end{aligned}$$
2. Let $v_o=u_0=\sum _{j=0}^{m}\alpha _j\,\phi _j(x)$, then from the first equation of (49) we define
  50 $$\begin{aligned} H+L=I. \end{aligned}$$
  From the 2nd equation of (49) we obtain
  51 $$\begin{aligned} \sum _{j=0}^{m}\alpha _j\,\phi _j(x)=f(x). \end{aligned}$$
  Approximating $\sin (x)$ by Chebyshev polynomials
  52 $$\begin{aligned} \sin (x)\approx \sqrt{\frac{\pi }{2}}\left( \frac{11}{24}\phi _1(x)-\frac{1}{48}\phi _3(x)\right) \end{aligned}$$
  and using first equation of (49) and taking account of (51) we get
  53 $$\begin{aligned} (H+L)\sum _{j=0}^{m}\alpha _j\,\phi _j(x)&= \sqrt{\frac{\pi }{2}}\left( -5 \phi _4(x)-\frac{11}{24}\phi _1(x)+\frac{1}{48} \phi _3(x)\right) . \end{aligned}$$
  Comparing the base of Chebyshev polynomial from the both sides of Eq. (53) the solutions are
  54 $$\begin{aligned} \alpha _4 = \sqrt{\frac{\pi }{2}}, \quad \alpha _1=\alpha _2=\alpha _3=0. \end{aligned}$$
3. Substituting (54) into Eq. (25) yields the exact solution
  55 $$\begin{aligned} \varphi (x)=\sqrt{1-x^2}\,(16 x^4-12x^2+1). \end{aligned}$$

Comparisons of Modified HPM and Chebyshev expansion Mahiub et al. (2011) is given in Table 2 for Eq. (48).

**Table 2:** Errors of approximate solutions for Eq. (48)

*x*	Mahiub et al. (2011)	Modified HPM
−1	0	0
−0.8	2.1 × 10^−10^	0
−0.6	4.0 × 10^−9^	0
−0.4	3.4 × 10^−9^	0
−0.2	2.3 × 10^−9^	0
0	2.0 × 10^−9^	0
0.2	2.3 × 10^−9^	0
0.4	3.3 × 10^−9^	0
0.6	4.0 × 10^−9^	0
0.8	2.7 × 10^−9^	0
1	0	0

### *Example 3*

Chen and Zhou (2011). Consider HSIE in the form

56 $$\begin{aligned} \frac{1}{\pi } =\!\!\!\!\!\!\int _{-1}^{1} \frac{\varphi (t)}{(x-t)^2}\, dt+\frac{1}{2\pi } \int _{-1}^{1} tx \,\varphi (t)\, dt = -8 x^3+\frac{17}{8} x-1,\quad -1<x<1, \end{aligned}$$

with exact solution $\varphi (x)=\displaystyle \sqrt{1-x^2}\,(1+2x^3)$.

Conditions of Theorem 8 are satisfied, therefore for HPM we choose initial guess as $u_0=\phi _1(x)$. Errors of numerical solution, computed for $N=\{5,10\}$ where *N* is a number of iteration, are given in Table 3.

**Table 3:** Errors of solutions for Eq. (56) solved by HPM

*x*	$N=5$	$N=10$
−0.9999	2.424502288 × 10^−8^	2.312185561 × 10^−14^
−0.901	6.701859769 × 10^−7^	6.391391532 × 10^−13^
−0.725	8.561715506 × 10^−7^	8.165088181 × 10^−13^
−0.436	6.727677283 × 10^−7^	6.416013033 × 10^−13^
−0.015	2.571605139 × 10^−8^	2.452473774 × 10^−14^
0.015	2.571605139 × 10^−8^	2.452473774 × 10^−14^
0.436	6.727677283 × 10^−7^	6.416013033 × 10^−13^
0.725	8.561715506 × 10^−7^	8.165088181 × 10^−13^
0.901	6.701859769 × 10^−7^	6.391391532 × 10^−13^
0.9999	2.424502288 × 10^−8^	2.312185561 × 10^−14^

To use modified HPM for solving Eq. (56), we do the following steps:

1. As usual we choose selective functions as $g_j(x)=\phi _j(x)$, $j=0,\ldots , m$ and kernel $L(x,t)=tx$ in Eq. (56) be approximated by projection kernel of the form $L_n(x,t)=\sum _{i=1}^{l}b_i(x)\phi _i(t)$. Again for this case $\tilde{L}u=Lu-L_nu \equiv 0$. Since $Cu\equiv 0, \ \ \tilde{L}u \equiv 0$ then $\tilde{S}=H+L$. Using (30) we have
  57 $$\begin{aligned} (H+L)v_0&=\sum _{j=0}^{m}\alpha _j\,\phi _j(x),\nonumber \\ (H+L)v_1&=f-\sum _{j=0}^{m}\alpha _j\,\phi _j(x). \nonumber \\ (H+L)v_k&=-\tilde{L}(v_{k-1}) \equiv 0, \, k=2,3, \ldots \end{aligned}$$
2. Again $v_k \equiv 0, \quad\, k=2,3, \ldots$ and by equating $v_1=0$ we have
  58 $$\begin{aligned} \sum _{j=0}^{m}\alpha _j\,\phi _j(x)=f(x). \end{aligned}$$
  using 1st equation of (57) and taking into account (58) yields
  59 $$\begin{aligned} (H+L)\sum _{j=0}^{m}\alpha _j\,\phi _j(x) = \sqrt{\frac{\pi }{2}}\left( -\phi _3(x)-\frac{15}{16}\phi _1(x)-\phi _0(x)\right) . \end{aligned}$$
  Comparing the base of Chebyshev polynomial from the both sides of Eq. (59) produce a system. Solutions of the system are
  60 $$\begin{aligned} \alpha _0=\sqrt{\frac{\pi }{2}},\quad\; \alpha _1=\frac{1}{2}\sqrt{\frac{\pi }{2}},\quad\;\alpha _2=0,\quad\;\alpha _3=\frac{1}{4}\sqrt{\frac{\pi }{2}}. \end{aligned}$$
3. Substituting (60) into Eq. (25) yields
  61 $$\begin{aligned} \varphi (x)=\sqrt{1-x^2}\,(1+2x^3). \end{aligned}$$
  which is identical with the exact solution.

Results are calculated by taking the maximum of absolute errors for Eq. (56). Comparison of the results between HPM, modified HPM and reproducing kernel Chen and Zhou (2011) shown in Table 4.

**Table 4:** Errors of solutions for Eq. (44)

*N*	Chen and Zhou (2011)	HPM	Modified HPM
5	6.8 × 10^−6^	8.57 × 10^−7^	0
10	5.2 × 10^−8^	7.8 × 10^−13^	0

### *Example 4*

Solve HSIE of the form

62 $$\begin{aligned} \frac{1}{\pi } =\!\!\!\!\!\!\int _{-1}^{1} \frac{1+2(t-x)}{(x-t)^2}\varphi (t)\, dt+\frac{1}{2\pi } \int _{-1}^{1} e^{2x} t^3 \,\varphi (t)\, dt = -16 x^4-40 x^3+4x^2+22 x+1+\frac{1}{32}e^{2x},\quad -1<x<1. \end{aligned}$$

The exact solution of Eq. (62) is $\varphi =\sqrt{1-x^2}(8x^3+4x^3-4x-1)$.

*Solution* For this example, the conditions of Theorem 8 does not hold. Therefore, HPM is not a reliable method to solve Eq. (62).

To obtain the approximate solutions of Eq. (62) by modified HPM (30), we do the following steps:

1. Approximate $L(x,t)=\dfrac{e^{2x}t^3}{2}$ by Chebyshev polynomials
  63 $$\begin{aligned} L_n(x,t)=\frac{e^{2x}}{16}(\phi _3(t)-2\phi _1(t))=L(x,t), \end{aligned}$$
  therefore $\tilde{L}_n\equiv 0$. Choose selective functions $g_j(x)=\phi _j(x)$, then from (30), we have
  64 $$\begin{aligned} (H+C)v_0&=\sum _{j=0}^{m}\alpha _j\,\phi _j(x), \end{aligned}$$
  65 $$\begin{aligned} (H+C)v_1&=f-\sum _{j=0}^{m}\alpha _j\,\phi _j(x). \end{aligned}$$
  66 $$\begin{aligned} (H+C)v_{k}&=-\tilde{L}(v_{k-1})\equiv 0, \end{aligned}$$
  with $$v_0=\sum _{j=0}^{m} b_j\,\phi _j(x)$$.
2. Since $v_k\equiv 0$, for $k\ge 2$ and approximating $e^{2x}$ into Chebyshev polynomials with 4 bases
  67 $$\begin{aligned} e^{2x}\simeq \sqrt{\frac{\pi }{2}}\quad \left( \frac{19}{12}\phi _0(x)+\frac{4}{3}\phi _1(x)+\frac{5}{8}\phi _2(x) +\frac{1}{6}\phi _3(x)-\frac{1}{24}\phi _4(x) \right) , \end{aligned}$$
  then substituting (67) into (65) and equating $v_1=0$ for $m=4$ yields
  68 $$\begin{aligned} b_0=\frac{19}{384}\,\sqrt{\frac{\pi }{2}},\quad b_1=\frac{25}{24}\,\sqrt{\frac{\pi }{2}},\quad  b_2=-\frac{507}{256}\,\sqrt{\frac{\pi }{2}}, \quad b_3 =-\frac{959}{192}\,\sqrt{\frac{\pi }{2}},\quad b_4=-\frac{767}{768}\,\sqrt{\frac{\pi }{2}}. \end{aligned}$$
3. From (64), we obtain the values of $\alpha _k$, $k=0,1,\ldots , 4$.
  $$\begin{aligned} \alpha _0=\alpha _1=\alpha _4=0, \quad \alpha _2=\alpha _3=\sqrt{\frac{\pi }{2}}. \end{aligned}$$
4. Substitute all values of $\alpha _i, i=0,\ldots , 4$ into (25), we have
  $$\begin{aligned} v_0=\sum _{k=0}^{4}\alpha _k \phi _k(x)=\sqrt{\frac{\pi }{2}}\phi _3(x)=8x^3+4x^3-4x-1. \end{aligned}$$
  Thus, we obtain the approximate solution in the form
  69 $$\begin{aligned} \varphi (x)=\sqrt{1-x^2}(8x^3+4x^3-4x-1) \end{aligned}$$
  which is same as exact solution. Modified HPM has zero error for solving Eq. (62).

### *Example 5*

Let us rewrite Eq. (4) in the form of

70 $$\begin{aligned} \frac{1}{\pi } =\!\!\!\!\!\!\int _{-1}^{1} \frac{2+tx(t-x)}{(x-t)^2}\varphi (t) \,dt+\frac{1}{\pi } \int _{-1}^{1} \left( \frac{1}{t+2}+\frac{1}{x+2}\right) \,\varphi (t) \,dt = f(x),\quad -1<x<1, \end{aligned}$$

where $$f(x)=\displaystyle -\frac{20\sqrt{3}}{2+x^2}-\frac{10 x^2}{x+2}(2-\sqrt{3}+x)+10\,(2-\sqrt{3})x+\frac{10}{3}(2\sqrt{3}-3)+\frac{10(2-\sqrt{3})}{x+2}$$.

The exact solution of Eq. (70) is $\varphi (x)=\displaystyle \sqrt{1-x^2}\frac{10}{x+2}$.

*Solution* Standard HPM is not suitable for solving the Eq. (70) as it is not satisfies the conditions in Theorem 8. For the modified HPM, we choose the selective functions $g_j(x)=\phi _j(x), \ \ j=0,\ldots ,m$. Approximating *L*(*x*, *t*) in Chebyshev polynomials form as follows

71 $$\begin{aligned} L_n(x,t)= x+\frac{1}{x+2}+\sum _{k=0}^{n}(-1)^k\frac{t^k}{2^{k+1}}. \end{aligned}$$

In this case $\tilde{L} \not = 0$, therefore the scheme (30) has the form

72 $$\begin{aligned} (H+C+L_n)v_0&=\sum _{j=0}^{m}\alpha _j\,\phi _j(x), \end{aligned}$$

73 $$\begin{aligned} (H+C+L_n)v_1&=-\tilde{L}+f-\sum _{j=0}^{m}\alpha _j\,\phi _j(x). \end{aligned}$$

To solve Eq. (70), we choose the collocation points, $x_i$ as the roots of $\phi (x)$ which is

74 $$\begin{aligned} x_i=cos\frac{(i+1)\pi }{n+2}, \quad i=0,1,\ldots n. \end{aligned}$$

Errors of $\varphi (x)$ using modified HPM for values of $m=\{6,26\}$ are presented in Table 5.

**Table 5:** Errors of solution for Eq. (70) solved by modified HPM

*x*	Modified HPM, $m=n=6$	Modified HPM, $m=n=6$
−0.9999	1.0851729 × 10^−4^	1.4040446 × 10^−10^
−0.901	3.5501594 × 10^−4^	1.8203460 × 10^−9^
−0.725	1.8899796 × 10^−4^	6.1943369 × 10^−8^
−0.436	3.0648319 × 10^−4^	2.6221447 × 10^−8^
−0.015	8.6784464 × 10^−5^	1.7385004 × 10^−8^
0.015	1.9992451 × 10^−5^	1.8524990 × 10^−8^
0.436	3.3916634 × 10^−1^	1.9700974 × 10^−8^
0.725	6.3326189 × 10^−5^	2.3479494 × 10^−8^
0.901	1.2745747 × 10^−4^	1.3403888 × 10^−8^
0.9999	3.3327100 × 10^−5^	3.5400390 × 10^−10^

From Tables 1, 2 and 4 show the comparison between the past method with HPM and modified HPM. It is clearly seen that Modified HPM gives more accurate results compare to the Chebyshev expansion method Mahiub et al. (2011), Bernstein polynomials approach Mandal and Bhattacharya (2007) and Reproducing Kernel method Chen and Zhou (2011).

Table 5 conclude that the modified HPM converges to the exact solution of Eq. (70) by increasing the number of collocation points *n* and number of selection functions *m*. It can also be seen that the convergence is achieved at all singular points *x* including the one which is close to the end points of the interval $[-1,1]$.

## Conclusion

In this work, the standard and modified HPM are used to find the approximate solution of the first kind HSIE. The theoretical aspect supported by the same numerical examples have shown the modified HPM gives better approximation than the standard HPM. Based on the examples, the modified HPM ables to handle the problem that can not be solved by standard HPM. Modified HPM is effective and reliable method for solving HSIE of the first kind of the form (4).
